# Editorial: Sustaining CO_2_ fertilization gains under water and nutrient stress in crop plants

**DOI:** 10.3389/fpls.2024.1375175

**Published:** 2024-02-21

**Authors:** Pengpeng Zhang, Narendra Kumar Lenka, Youhong Song

**Affiliations:** ^1^School of Agronomy, Anhui Agricultural University, Hefei, Anhui, China; ^2^Division of Soil Physics, Indian Council of Agricultural Research (ICAR)-Indian Institute of Soil Science, Bhopal, Madhya Pradesh, India; ^3^Centre for Crop Science, Queensland Alliance for Agriculture and Food Innovation, The University of Queensland, Brisbane, QLD, Australia

**Keywords:** climate change, nitrogen limitation, physiological response, photosynthesis, soil nutrient pool

Confronted with issues of climate change, food security in the framework of sustainable agriculture is becoming increasingly challenging. According to the IPCC’s latest AR6 Synthesis Report, ambient carbon dioxide (CO_2_) concentrations have increased from the pre-industrial level of 280 ppm (by volume) to approximately 410 ppm (by volume) at present (IPCC, 2023). At the same time, global surface temperature has increased by 1.1°C between 2011 and 2020 as compared to the 1850-1900 period and additionally, the complexities of altered precipitation patterns and climate extremes such as drought stress have emerged. Since CO_2_ is a primary raw material in the process of leaf photosynthesis, increased atmospheric CO_2_ levels have the potential to stimulate plant growth and development and thus enhance crop productivity. Yield has been reported to increase by up to 18% in cereals, legumes, and root crops under non-stress conditions in free-air CO_2_ enrichment (FACE) experiments (Ainsworth and Long, 2021). The effects of increasing atmospheric CO_2_ concentrations on carbon, water, and nitrogen metabolism are primarily determined by changes in stomatal behavior and photosynthetic efficiency in plants. The biophysical and physiological responses of crop plants to elevated CO_2_ in a stress-free environment have been well studied. However, the combined effects of CO_2_ and temperature increases under nutrient and water-limited conditions on plant responses, soil systems, and nutrient and water use efficiency will be key to future agriculture (Lenka and Lal, 2012; Lenka et al., 2021a). These crucial research gaps have large socio-economic ramifications, due to the scarcity of water and the depletion of soil resources for future agriculture. Harnessing the benefits of increased CO_2_ for greater crop productivity is essential, particularly in the face of mounting challenges such as water scarcity and nutrient limitations. However, the extent to which the benefits of CO_2_ fertilization can be realized under water and nutrient stress in warming situations is not fully understood. In this context, this Research Topic brings together discussions on multifaceted issues regarding CO_2_ fertilization gains in crop plants, including heat stress (Liu et al.), drought stress (Cao et al.), nitrate assimilation and photorespiration (Krämer et al.), reproductive success (Li et al.), and grain quality (Jo et al.).

With the increase in ambient CO_2_ concentration, heat, and drought stress are projected to become more frequent and more severe, posing a major challenge to sustainable crop production (Harrison et al., 2014; Lobell et al., 2014; Yuan et al., 2023). Liu et al. examined the combined effects of elevated CO_2_ concentration and temperature on maize growth and development, indicating that the strongest effect of CO_2_ fertilization on maize plants could be achieved at the highest temperature (37/31°C) due to higher leaf soluble sugars, lower stomatal aperture, and substantially compromised leaf transpiration. As such, the CO_2_ fertilization effect can be realized by adjusting leaf anatomy, stomatal traits, and soluble sugar content as a function of growth temperature. Reduced leaf stomata are often associated with reduced crop water consumption. Under non-drought stress conditions, Cao et al. found that CO_2_ elevation exclusively increased leaf photosynthesis and assimilate accumulation in wheat, while concurrently decreasing stomatal conductance and water consumption in both wheat and maize. This dual effect led to a notable improvement in water use efficiency (WUE), with a 27.82% increase in maize and a greater boost of up to 49.86% for wheat. Under the combination of elevated CO_2_ and drought stress, improved WUE was only found in wheat, although maize plants showed greater drought resistance demonstrating the differential behavior of C3 and C4 plants to elevated CO_2_ and progressive water deficits. Therefore, specific water management strategies should be developed in the future to maximize crop WUE for different species in a combination of elevated CO_2_ and drought stress environments.

The positive effect of elevated CO_2_ on WUE may be further enhanced under higher nitrogen availability, as nitrogen assimilation in plants regulates the CO_2_ fertilization effect (Ainsworth and Long, 2021; Lenka et al., 2021b). However, the reasons for elevated CO_2_ leading to a reduction in nitrogen assimilation are unclear, although an effect of reduced photorespiration at elevated CO_2_ has been suggested. To investigate the role of photorespiration in nitrogen assimilation, Krämer et al. used a mutant defective in peroxisomal hydroxy-pyruvate reductase (*hpr1-1*) that is impaired in photorespiratory turnover, and found that photorespiration stimulated nitrogen assimilation. However, nitrate assimilation was not reduced at elevated CO_2_, pointing to a dilution of nitrogen-containing compounds by assimilated carbon at elevated CO_2_. This study confirms a close link between photorespiration and nitrogen assimilation and fills the knowledge gap on how plant N assimilation is affected by elevated CO_2_. The majority of studies have reported that elevated CO_2_ leads to an increase in carbohydrate content, which dilutes mineral and protein concentrations in plant tissues (Taub et al., 2008; Ainsworth and Long, 2021). To investigate distinct changes in fennel growth characteristics and phytonutrient content under elevated CO_2_, Jo et al. performed transcriptome analysis and identified CO_2_-responsive differentially expressed genes involved in photosynthesis, Karrikin response, and flavonoid biosynthesis, suggesting that elevated CO_2_ has a strong effect on plant improvement.

Li et al. summarized the major limitations of leaf source and kernel sink in maize under drought stress and pointed out that the loss in grain yield is mainly due to reduced kernel set. Utilization of CO_2_ fertilization through improved photosynthesis and greater reserve remobilization is a key strategy for enhancing reproductive drought tolerance. To capitalize on this potential, the authors proposed a series of strategies to adapt to rising CO_2_ concentrations, including optimizing planting methods, mining natural genetic variation, exploiting advanced genetic engineering.

Overall, this Research Topic is dedicated to deciphering the intricate interaction of physiological, biochemical, and genetic factors that underlie plant responses to elevated CO_2_, shedding light on unlocking the complexity of such interaction. In addition, this Research Topic explores innovative strategies and technologies to mitigate the detrimental effects of water and nutrient stress on crop yields in a changing climate. Consequently, the Research Topic includes a compendium of knowledge, insights, and solutions that seek to bridge the gap between the promise of CO_2_ fertilization and the reality of sustainable crop production.

## Author contributions

PZ: Writing – original draft, Writing – review & editing. NL: Conceptualization, Writing – original draft, Writing – review & editing. YS: Conceptualization, Writing – original draft, Writing – review & editing.
